# Abdomen aigu sur une torsion de rate ectopique: à propos d'un cas

**Published:** 2012-04-04

**Authors:** Elhattabi Khalid, Bensardi Fatimazahra, Lefriyekh Rachid, Fadil Abdelaziz, Lahkim Mohamed, Benissa Nadia, Khaiz Driss, Berrada Saad, Zerouali Ouariti Najib

**Affiliations:** 1 Service des urgences chirurgicales viscérales – CHU Ibn Rochd, Casablanca, Maroc

**Keywords:** Rate, abdomen aigu, scanner abdominal, chirurgie, Maroc

## Abstract

La rate ectopique est une entité rare dont l'incidence est mal connue. La torsion de son pédicule est une complication grave, qui doit bénéficier d'un diagnostic précoce; évitant ainsi la splénectomie surtout chez des patients jeunes. Nous rapportons le cas d'une patiente âgée de 39 ans; admise aux urgences dans un tableau d'abdomen aigu chirurgical, l'examen clinique, l’échographie et la tomodensitométrie abdominale ont contribué au diagnostic de torsion d'une rate ectopique en position hypogastrique, la splénectomie a été réalisée devant la découverte peropératoire de taches de nécrose sur la rate tordue. Le diagnostic de torsion d'une rate ectopique doit être suspecté devant l'association d'un abdomen aigu et la palpation d'une masse abdominale. L’échographie et la tomodensitométrie abdominale confirment le diagnostic. Le traitement est chirurgical; il consiste en une splénopexie qui est plus approprié. En présence de nécrose, la splénectomie doit être réalisée aussi bien par voie laparoscopique que par laparotomie.

## Introduction

La rate ectopique est une curiosité anatomique résultant d'une anomalie congénitale ou acquise des moyens de fixité de la rate, ce qui lui permet un certain vagabondage, ainsi nous pouvons la palper dans tous les quadrants de l'abdomen en fonction de la longueur de son pédicule. La torsion de son pédicule est une complication grave, qui doit bénéficier d'un diagnostic précoce; évitant ainsi la splénectomie surtout chez des patients jeunes.Le but de ce travail est de rapporter une observation rare d'une torsion de rate ectopique révélée par un tableau d'abdomen aigu chirurgical avec une revue de la littérature.

## Patient et observation

Mme LF, âgée de 39 ans, présentant depuis cinq ans des douleurs abdominales paroxystiques spontanément résolutives, et qui a été admise aux urgences pour des douleurs abdominales intenses péri-ombilicales apparues deux jours avant son hospitalisation d'installation brutale avec des vomissements bilieux, sans trouble du transit, ni hémorragie digestive extériorisée. A l'examen: la patiente était stable sur le plan hémodynamique et respiratoire avec un fébricule à 38,2°. L'abdomen était légèrement distendu avec une défense péri-ombilicale et hypogastrique et présence d'une masse siégeant à cheval de la région ombilicale et hypogastrique mesurant 15/7cm douloureuse, de consistance ferme, mobile et mate à la percussion, les touchers pelviens n'ont pas perçu le pôle inferieur de la masse. L’échographie abdominale était gênée par les gaz digestifs, néanmoins elle a suspecté la torsion d'une rate ectopique devant la vacuité de la loge splénique et la présence d'une masse hypogastrique non vascularisée au doppler. La tomodensitométrie abdomino-pelvienne avait montré un aspect évocateur d'une rate ectopique hypogastrique compliquée très probablement d'une torsion pédiculaire devant l'absence de prise de contraste.

**Figure 1:**
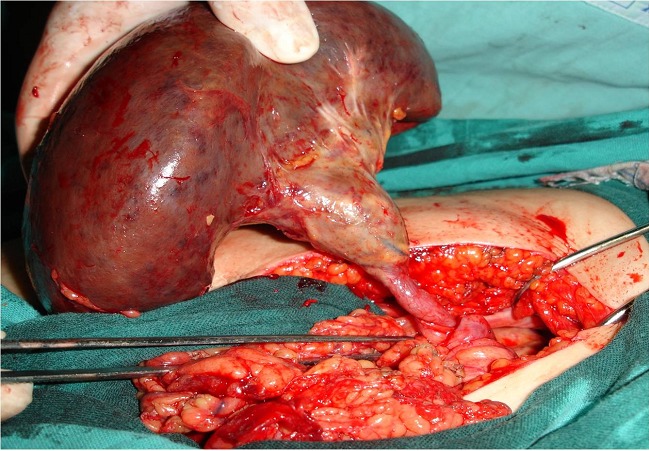
vue per-opératoire de la rate hypogastrique avec torsion pédiculaire et des taches de nécrose.

La patiente a été opérée après avoir reçu la vaccination anti- pneumococcique et une antibioprophylaxie (association Amoxicilline et acide clavulanique), par une laparotomie médiane à cheval sur l'ombilic. L'exploration a retrouvé une rate augmentée de taille, libre, avec absence totale d’épiploon gastro-splénique et pancréatico-splénique et du ligament spléno-colique; située en position hypogastrique; violacée avec des taches de nécrose centimétriques et torsion pédiculaire en deux tours de spire (Figure 1). La loge splénique était vide occupée par les anses intestinales avec absence de rate surnuméraire. Une splénectomie a été effectuée. Les suites postopératoires étaient simples. La durée totale d'hospitalisation était de quatre jours. L'examen anatomo-pathologique de la pièce opératoire a retrouvé des foyers de nécrose ischémique splénique.

## Discussion

La rate ectopique ou rate baladeuse se définit comme une migration de la rate vers n'importe quelle région de la cavité péritonéale suite à une anomalie congénitale ou acquise de ses moyens de fixité. C'est une entité rare qui est souvent rencontrée chez des femmes en période d'activité génitale [1–5].Sur le plan étiologique, elle peut être congénitale ou acquise. La forme congénitale est due à un défaut d'accolement du mésogastre postérieur avec allongement du pédicule splénique; ce qui confie à la rate une certaine liberté dans la cavité abdominale. Quant à la forme acquise, plusieurs facteurs ont été incriminés; comme la distension gastrique; la splénomégalie; l'hyperlaxité abdominale; le traumatisme abdominal et la grossesse. Dans notre observation, l'absence totale d’épiploon gastro-splénique et pancréatico-splénique et du ligament spléno-colique confirme bien l'origine congénitale de cette rate ectopique [2].

Sur le plan clinique, la rate ectopique peut rester asymptomatique et n'est découverte que fortuitement lors d'un examen abdominal ou d'un bilan d'imagerie (échographie ou tomodensitométrie abdominales) fait pour une autre indication[1, 5–8].Elle peut aussi se manifester par des douleurs abdominales intermittentes témoignant de crises de torsion et de détorsion spontanée; comme c’était le cas de notre patiente qui rapportait des crises douloureuses paroxystiques spontanément résolutives depuis cinq ans et qui n'ont jamais motivées de consultation. La torsion de la rate ectopique favorisée par sa mobilité, son poids et la longueur de son pédicule peut être irréversible et se manifester par un abdomen aigu chirurgical; où la douleur abdominale vient au premier plan; associée parfois à des nausées; des vomissements et de la fièvre. La palpation de l'abdomen recherche des signes péritonéaux et une masse abdominale ou pelvienne [6, 7, 9].L'association de ce tableau d'abdomen aigu à une masse abdominale mobile doit faire évoquer le diagnostic de torsion de rate ectopique au même titre que la torsion de masse ovarienne, l'abcès abdominal ou le volvulus sur tumeur du grêle; et faire indiquer un bilan d'imagerie pour faire le diagnostic.

L’échographie abdomino-pelvienne permet d'affirmer le diagnostic de torsion de la rate ectopique devant la vacuité de la loge splénique et la mise en évidence d'une masse abdominale rappelant l’échostructure splénique; l'absence de vascularisation au doppler est un argument en faveur de la torsion [4, 7, 9].

La tomodensitométrie abdomino-pelvienne confirme le diagnostic de la torsion de la rate ectopique en montrant une loge splénique vide avec une masse abdominale ou abdomino-pelvienne rappelant l'aspect de la rate et ne prenant pas le produit de contraste après injection intraveineuse [3–5, 7, 9]. Le pédicule tordu peut se voir au scanner sous forme de Tourbillon représentant les tours de spire [9].

Une fois le diagnostic de cette complication est posé; l'exploration chirurgicale s'impose. Elle confirme le diagnostic et permet d'adapter le traitement. En effet, en l'absence de nécrose splénique, la détorsion peut être réalisée avec une splénopexie permettant de fixer la rate dans sa position anatomique normale. En cas de nécrose splénique; il faut réaliser la splénectomie. Ces gestes sont faisables aussi bien par voie conventionnelle que laparoscopique [1, 4, 5, 7–10].

## Conclusion

Le diagnostic de torsion d'une rate ectopique doit être évoqué devant l'association d'un tableau d'abdomen aigu chirurgical à une masse abdominale. L’échographie et la tomodensitométrie abdomino-pelviennes sont suffisantes pour poser le diagnostic et indiquer un traitement chirurgical avant l'installation de l'infarctus splénique. La splénopexie doit être réalisée en l'absence de nécrose surtout chez le sujet jeune. La laparoscopie comme voie d'abord mini invasive doit être privilégier tant qu'elle est possible.
